# Theory of the Kinetics of Chemical Potentials in Heterogeneous Catalysis

**DOI:** 10.1002/anie.201101459

**Published:** 2011-06-29

**Authors:** Jun Cheng, P Hu

**Affiliations:** * School of Chemistry and Chemical Engineering, The Queen's University of BelfastBelfast BT9 5AG (UK)

**Keywords:** catalysts, chemical potentials, heterogeneous catalysis, reaction kinetics

Catalysis is of paramount importance in our daily life. In particular, heterogeneous catalysts which speed up reactions on their solid surfaces play a vital role in a wide range of industries, such as petroleum, energy, and environment-related industries. There is no doubt that rational design of new catalysts is a major endeavor in chemistry. A key to achieve this goal is reaction kinetics, bridging the gap between microscopic elementary chemical reactions and macroscopic performance of catalysts. Although great progress in understanding reaction kinetics has been made, rational design of new catalysts remains one of the profound challenges. Here, we present a new formulism of reaction kinetics at surfaces in terms of the involved chemical potentials, which simplifies the reaction kinetics significantly. Furthermore, within this formulism we propose a new approach of searching for new catalysts. The effectiveness and universality of this theory are discussed.

There have been several major related developments in this field. First, density functional theory (DFT) approaches have been developed to such a level that the barriers of elementary steps are determined routinely. Many total-energy profiles of reaction systems from DFT calculations were reported. Second, with the energy profiles from first-principles calculations in hand, kinetic information, such as reaction rates and coverages of surface intermediates, are obtained by kinetic Monte Carlo simulations^1^, ^2^ and microkinetic calculations.^3^ Third, the linear relationship between the reaction barrier and the reaction-enthalpy change, the so-called Brønsted–Evans–Polanyi (BEP) relations,^4^ was found to be followed by a wide range of surface reactions.^5^–^8^ Based on the BEP relations, some successful examples of catalyst design from first-principles calculations, that is, the activities were plotted against the adsorption energies of key intermediates (see, for example, Refs. 9, 10), were reported.^11^, ^12^ Fourth, Nørskov and co-workers^9^, ^13^ observed that for many surface reactions on the best catalysts the adsorption energies of key intermediates locate in a small window of −2 to −1 eV (negative energy means the adsorption is exothermic). Significantly, this energy window appears to be universal. This is somewhat surprising, considering that the catalytic systems are very different and each of them possesses unique, complicated kinetics.

However, the computation of the reaction barrier of each elementary step is time-consuming, and moreover the understanding of the catalytic systems from the total-energy profiles obtained consequently is not straightforward, let alone rational design of new catalysts. As a result, new catalysts are traditionally developed using experimental trial-and-error methods. Therefore, to have better approaches, in particular better kinetic theories directed towards the design of catalysts, is essential to further develop the subject. Here, we introduce chemical potentials, which are widely used in electrochemistry under the name of electrochemical potential, to surface-catalytic reactions to reformulate the reaction kinetics. It will be manifested below that the chemical potentials of surface intermediates implicitly take into account surface coverages and temperature effects, and therefore reveal more chemical meanings. More importantly, we show that chemical potentials of surface intermediates can be used as a guide in searching for new catalysts without detailed kinetic analyses, and the interesting observation of the universal energy window can be understood using our approach.

The basis of our approach is the expression of the chemical potential of a surface species in the Langmuir adsorption paradigm [Eq. (1); see the Supporting Information for the derivation]:^14^–^16^


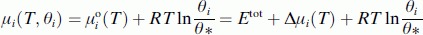
(1)

where *θ_i_* and θ_*_ are coverages of adsorbed species *i* and free sites * on a surface, respectively, and 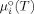 is the standard chemical potential of species *i* at temperature *T* and is readily obtained from the total energy *E*^tot^, routinely computed using DFT, at 0 K with a small thermal correction term Δ*µ_i_*(*T*).

Based on Equation (1), many expressions for the microkinetics, for example, the reaction rate and reversibility, can be reformulated by using only chemical potentials. The formulations for some typical elementary surface reactions are given in the Supporting Information. To illustrate our method, we apply a simple two-step kinetic model consisting of adsorption [Eq. (2)] and desorption [Eq. (3)] processes, which captures the essence of many heterogeneous catalytic reactions,^8^ where R and P are reactant and product in the gas phase, and I and * are surface intermediate and free site, respectively.


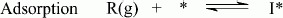
(2)


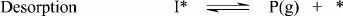
(3)

Following our formulation, we can rewrite the kinetic equations of reactions (2) and (3) as Equations (4)–(7), where *r*_ads_ and *r*_des_ are the reaction rates of the adsorption and desorption processes, respectively, *z*_ads_ and *z*_des_ are the reversibilities of the adsorption and desorption processes,^17^ respectively, *µ*_R_, *µ*_I_, and *µ*_P_ are the chemical potentials of the reactant, the surface intermediate, and the product, respectively, and 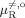 and 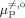 are the standard chemical potentials of the transition states (TS) of adsorption and desorption, respectively. At the steady state, *r*=*r*_ads_=*r*_des_, where *r* is the overall reaction rate.


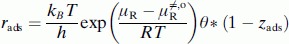
(4)


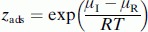
(5)


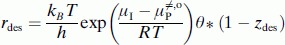
(6)


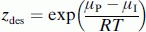
(7)

From Equations (4)–(7), we obtain Equation (8), where 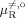 and 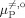 are, according to the BEP relation, linearly related to 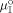, which is an intrinsic property of the catalyst surface^5^–^8^ and independent of reaction conditions. Thus, solving Equation (8) with the condition of conservation of the surface site *θ*_I_+θ_*_=1, we calculate *θ*_I_ and θ_*_, as well as the overall reaction rate. If treating 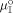 (equivalent to the adsorption energy) as a variable, the overall reaction rate *r* will be a function of adsorption energy, giving rise to a typical volcano curve.^9^, ^10^


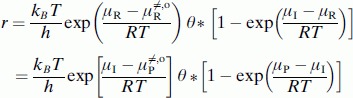
(8)

What can we learn from the kinetics of chemical potentials compared to traditional kinetic equations for catalytic reactions? First, the equations shown above contain some useful chemical insights. By plotting the reaction profiles of the chemical potentials (the gray curve in Figure 1), we obtain a deeper understanding of surface reactions compared to the traditional reaction profiles currently used, in which only total energies are considered without thermal correction and concentration terms (the black curve in Figure 1). Useful kinetic information can hardly be obtained from the conventional reaction profiles of total energies by DFT calculations, whereas in the profile of chemical potentials the reactants, intermediates, and products along the reaction coordinate must decrease step by step [Eq. (9)].


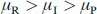
(9)

**Figure 1:**
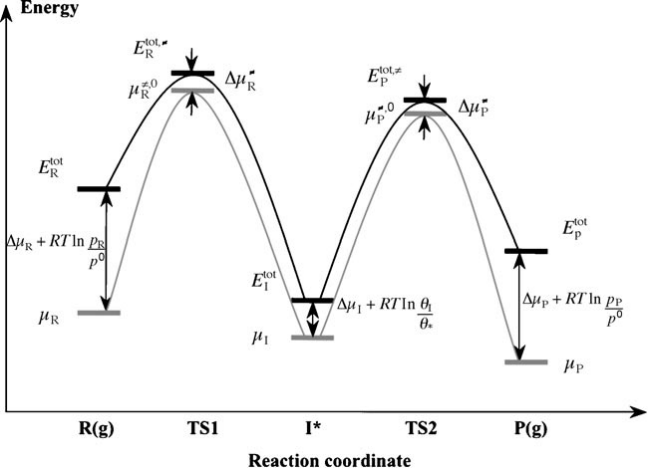
Energy diagram of a model for heterogeneous catalytic reactions. The black curve stands for the profile of total energies calculated from DFT, and the gray curve represents the profile of chemical potentials. TS1 and TS2 are the transition states (TSs) of adsorption and desorption, respectively. *E*^tot^ is the total energy, and *µ* is the chemical potential (subscript R, I and P refer to reactant, intermediate, and product). 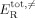 and 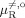 are the total energy and standard chemical potential of the TS of adsorption, respectively, 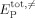 and 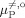 have the same meanings for the TS of desorption. The correction of the chemical potential because of the temperature effect is given by Δ*µ*. The thermal corrections for gaseous molecules (Δ*µ*_R_ and Δ*µ*_P_) are quite large because of large entropy effects, whereas the corrections for surface species (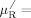, Δ*µ*_I_ and 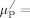) are much smaller. *R* *T*ln(*θ_i_*/θ_*_) is the coverage-dependent term in the expression of the chemical potential of surface species [see Eq. (1)], and likewise *R* *T*ln(*p*/*p*^o^) is the pressure-dependent term for gaseous molecules [see Eq. (S1) in the Supporting Information]. Unlike intermediate state, the standard chemical potentials for the TSs appear in the profile of chemical potentials.

This equation can be generalized to any sequential multistep reaction system, namely 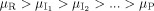. Its significance will be revealed later. Second, the magnitude of the decrease of each step, which is related to the reversibility according to Equations (5) and (7), indicates the thermodynamic driving force for the step. Third, the heights of the TSs with respect to the reactant states (the barriers of the chemical potentials) are direct measures of the reaction rates, unlike the barriers of total energies in which the entropic effects and coverages are not taken into account. This is best manifested by the fact that for adsorption processes the sole use of the barriers of total energies as measures will significantly overestimate the reaction rates because of the lack of large negative entropic effects.

There are some significant implications in the above results for understanding heterogeneous catalysis. First, if the standard chemical potential of a TS of a prior step is smaller than the later step, that is, 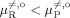, then *µ*_R_≍*µ*_I_ is approximately satisfied (see the Supporting Information), indicating that the former step reaches a quasi-equilibrium at steady states. This result can be readily extended to other sequential elementary reactions, such as the multistep hydrogenation reaction C+4 H→CH_4_ in the CO hydrogenation on metal surfaces.^18^, ^19^ For a series of sequential reactions if the last step has the highest chemical potential at the TS, the previous steps can be approximately treated as being in quasi-equilibrium, and the last step is the rate-determining. Second, for a given reaction condition and a catalyst surface, the levels of the chemical potentials of reactant and product (*µ*_R_ and *µ*_P_) and TSs (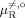 and 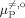) are fixed in a reaction profile of chemical potentials. However, the chemical potential of the surface intermediate (*µ*_I_) consists of two terms, 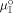 and *R* *T*ln(*θ_i_*/θ_*_), where 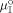 is invariant for a given catalyst and *R* *T*ln(*θ_i_*/θ_*_) is the coverage-dependent term. Namely, *µ*_I_ is varied around the 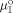 by θ_*_, considering that θ_*_ is a variable whereas *θ*_I_ is not an independent variable because of *θ*_I_+θ_*_=1 in our two-step model (but generally, *θ*_I_+θ_*_≍1 if there are more than one intermediate and intermediate I is the main one). Upon approaching a steady state, however, the level of *µ*_I_ must reside between *µ*_R_ and *µ*_P_ to satisfy Equation (8).

Now we are in a position to illustrate an important application of our chemical potential kinetics theory for searching for new catalysts. In the Supporting Information, using a simple kinetic model we show that the coverage of free sites θ_*_ on the surface can be derived to be around 10^−1^ monolayer (ML) for the optimal catalysts, which is supported by experimental results: When catalytic reactions take place on good catalysts, θ_*_ is usually in the order of magnitude of 10^−1^ to 10^−2^ ML at steady states. For example, kinetic analyses showed that θ_*_ is around 0.08 ML for hydrogenation of isobutene on Pt,^17^ and around 0.01 ML for the synthesis of ammonia on Fe and Ru catalysts.^7^, ^17^ This is also consistent with a general consensus in the field: 1) If θ_*_ is low, it is usually a sign of blockage of surface sites, leading to low activities. This often happens when the surface–adsorbate bonding interaction is too strong. 2) If θ_*_ is high and approaches 1 ML, it is difficult for molecules to adsorb on the surface. Namely, the surface is too inert to catalyze the reaction.

Since θ_*_ is about 10^−1^ to 10^−2^ ML on the best catalysts and θ_*_ and *θ*_I_ are related to each other because of the conservation of surface sites, the magnitude of the coverage-dependent term *RT* ln(*θ_i_*/θ_*_) in Equation (5) has to lie in a small range, about 0.1–0.2 eV at 500 K, and is defined as *ε*. In other words, for good catalysts [Eq. (10)].


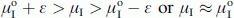
(10)

Combining Equations (9) and (10), we reach the key relation [Eq. (11)] for searching for good catalysts.


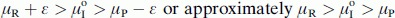
(11)

In principle the coverage-dependent term *RT* ln(*θ_i_*/θ_*_) varies in the range from −∞ to +∞ and approaches the infinity limits when the surface coverage is extremely low (no adsorption) and high (1 ML). Thus, *µ*_I_ can be changed by θ_*_ to locate anywhere in the diagrams of chemical potentials no matter where 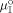 is. However, upon approaching the two limits the total reaction rates will be reduced dramatically because of the inertness of the surface or blockage of surface sites. For good catalysts *RT* ln(*θ_i_*/θ_*_) should be small, and hence *µ*_I_ is mainly determined by 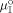. Equation (11) may partially justify the assumption of a downhill requirement for the free-energy diagrams in the model for electrocatalytic reactions reported by Nørskov, Rossmeisl, and co-workers.^20^

Then, the searching procedure of catalysts, as illustrated in Figure 2, can be devised: First, based on the gas-phase energetics of an overall reaction under a certain reaction condition (i.e. temperature and pressure) which can be very easily obtained, determine the positions of the chemical potentials of the reactants and products in the diagram of chemical potentials, which approximately establishes the upper and lower boundaries of the standard chemical potentials of the key intermediates on the surface (zone 1 in Figure 2). Second, according to Equation (11), we slightly relax the boundaries (e.g. by 0.2 eV) and search for the appropriate catalyst surfaces which are able to offer the standard chemical potentials of the surface intermediates lying between the relaxed boundaries (zone 2 in Figure 2). As can be seen from Figure 2, 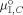 and 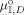 satisfy the requirement and the corresponding catalysts are most likely good catalysts. However, 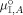 and 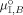 are too high or too low to locate in the zone even including the small variation because of the coverage term, and hence the corresponding catalysts cannot be good catalysts. This approach is extremely simple, but its effectiveness and universality can be seen later.

**Figure 2:**
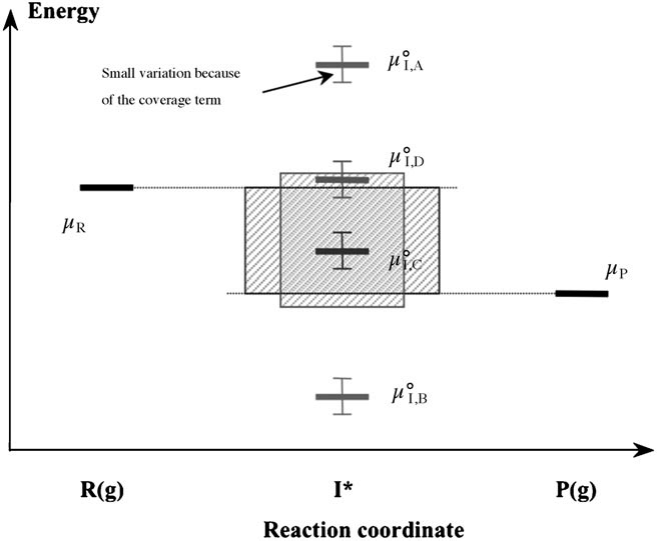
Searching for good catalysts by means of the involved chemical potentials. The chemical potentials of reactant and product (*µ*_R_ and *µ*_P_) set the boundaries for the chemical potential of the surface intermediate (*µ*_I_, zone 1). On good catalysts, this zone can only be slightly relaxed for the standard chemical potential of the surface intermediate (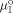, zone 2). Thus, surfaces of catalysts related to 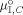 and 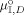 are very likely to be good catalysts, whereas surfaces related to 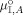 and 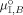 cannot be good catalysts.

Since the standard chemical potential of a key surface intermediate 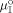 consists of a dominating total-energy term 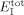, related to the adsorption energy on the surface and a small thermal correction term Δ*µ*_I_(*T*) according to Equation (1), a good catalyst must have an appropriate binding energy of the key intermediate lying between the boundaries set by the chemical potentials of the reactant and the product. This coincides with the idea of adsorption-energy windows proposed by Nørskov and co-workers, stating that the optimal catalyst should be one with an chemisorption energy of the adsorbate in the range of −2 to −1 eV.^9^, ^13^

Here, we use the ammonia synthesis as an example to elaborate our method. According to Equation (9), we can obtain the boundaries for the chemical potential of adsorbed N [Eq. (12), see the Supporting Information for details].


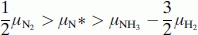
(12)

Substituting Equation (10) and applying experimental gas-phase energetic data under typical reaction conditions (H_2_ 75 bar, N_2_ 25 bar, NH_3_ 1 bar, 673 K), we can rewrite Equation (12) into Equation (13), where 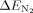 is the dissociative adsorption energy of N_2_.


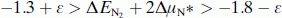
(13)

Correcting the small term 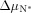 as well as the zero-point energy (ZPE),^21^ we have Equation (14).


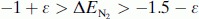
(14)

Taking *ε* as 0.2 eV as suggested above, we will have adsorption energies of N_2_ in the range of around −1.7 to −0.8 eV for optimal catalysts, which is in good agreement with the observed energy window (around −2 to−1 eV).^9^, ^13^ This agreement is extraordinary: In predicting the optimal range of the adsorption energy, we only use the data from gas-phase reaction energetics, without recourse to detailed DFT calculations on surfaces except the small corrections of 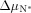 and the ZPE.

A universal upper boundary of optimal adsorption energy can be estimated and understood using our formulation. Heterogeneous catalytic reactions start with adsorption of reactants on surfaces, which must satisfy *µ*_R_>*µ*_I_. Applying Equation (1) and ignoring small concentration and thermal correction terms, we can obtain −*T* *S*_R_>Δ*E*_R_, where *S*_R_ is the entropy of the reactant in the gas phase and Δ*E*_R_ is the adsorption energy of the reactant. Therefore, the upper boundary is mainly set by the entropy of the reactant, that is, the adsorption energy of the reactant must be strong enough to outweigh the decrease in entropy upon adsorption. Bearing in mind the fact that many small molecules have the entropies of around 200 J mol^−1^ K (e.g. N_2_ in the ammonia synthesis, CO in its hydrogenation reaction and O_2_ in the CO oxidation), we reach the upper boundary of adsorption energy of around −0.6 to −1.0 eV at a typical temperature range of 300–500 K. This estimate also agrees with the upper boundary of the observed energy window.^9^, ^13^ The lower boundary of optimal adsorption energy may be not that obvious as the upper boundary, but should readily be obtained, as already shown in the ammonia synthesis.

Finally, we wonder why this method works by considering only reaction energetics (i.e. chemical potentials of reactants and products and standard chemical potential of key intermediates) without taking reaction kinetics into account (reaction barriers are not involved). In fact, the kinetic information is implicitly folded by the fact that the coverage-dependent term *R* *T*ln(*θ_i_*/θ_*_) in Equation (1) is small for good catalysts.

In summary, here we have applied chemical potentials to catalytic reactions on surfaces; reaction kinetics of surface processes, for example, the reaction rate and reversibility, has been reformulated in terms of the involved chemical potentials. The total energy profiles of surface reactions usually computed from DFT simulations can be readily converted to profiles of chemical potentials. The new formulation is simple but powerful to understand surface reactions both thermodynamically and kinetically. We have estimated with approximations that for many catalytic reactions the coverage of free site on surfaces of the best catalysts is usually in a medium range. Combining this simple, but important result and our formulation, a procedure in searching for good catalyst has been proposed. Our formulation has also provided the explanation of the universality of the adsorption-energy window in heterogeneous catalysis. We have further shown our method by using the ammonia synthesis as an example. Our method is not only able to predict the range of optimal adsorption energies, in agreement with reported values, but it also is very simple without the need of extensive calculations of reaction barriers and detailed kinetic analyses.

## Supplementary material

Detailed facts of importance to specialist readers are published as ”Supporting Information”. Such documents are peer-reviewed, but not copy-edited or typeset. They are made available as submitted by the authors.
