# Supplementary material for: Theory of the Kinetics of Chemical Potentials in Heterogeneous Catalysis
Source: Angew Chem Int Ed Engl. 2011 Jun 29;50(33):7650–4. doi: 10.1002/anie.201101459 (PMC3625742; doi:10.1002/anie.201101459)
Supplement: Supplementary file 1 [file anie0050-7650-SD1.pdf]

Angewandte Chemie
Eine Zeitschrift der Gesellschaft Deutscher Chemiker

Supporting Information

© Wiley-VCH 2011

69451 Weinheim, Germany

Theory of the Kinetics of Chemical Potentials in Heterogeneous Catalysis

Jun Cheng and P. Hu**

anie_201101459_sm_miscellaneous_information.pdf

1 Chemical potentials of species in the gas phase and on surfaces

We start with the chemical potential of an ideal gas. The dependence on its partial pressure p at a given temperature T can be expressed as

$$\mu(T,p)=\mu^o(T,p^o)+RT\ln\frac{p}{p^o} \tag{S1}$$

where $\mu^o(T,p^o)$ is the chemical potential at the standard pressure p^o (1 bar). Since the interaction between molecules is ignored in the ideal gas model, the chemical potential is equal to molar Gibbs free energy (i.e. $\mu=G_m=G/N$). If choosing the temperature of 0 K as a reference, Eq. (S1) can be further broken into:

$$\begin{aligned}\mu(T,p)&=\mu^o(0K,p^o)+[H_m(T,p^o)-H_m(0K,p^o)]-TS_m(T,p^o)+RT\ln\frac{p}{p^o}\\&=\mu^o(0K,p^o)+\Delta\mu(T,p^o)+RT\ln\frac{p}{p^o}\end{aligned} \tag{S2}$$

in which we have used the relations $G_m=H_m-TS_m$ and $S_m(0K)=0$ among the molar Gibbs free energy G_m, the molar enthalpy H_m and the molar entropy S_m. The difference between $\mu^o(T,p^o)$ and $\mu^o(0K,p^o)$ is folded in the term of $\Delta\mu(T,p^o)$, referring to the thermal correction of chemical potential with reference to zero K. The enthalpic correction is usually small and hence $\Delta\mu(T,p^o)$ is dominated by the entropy part $-TS_m(T,p^o)$. Since at zero K entropy is zero, $\mu^o(0K,p^o)$ is equal to the zero point energy (ZPE) corrected total energy of an isolated molecule (E^{tot}), which can be directly obtained from DFT calculations. In the following, we will drop the labels of temperature and pressure, and use μ^o instead of $\mu^o(T,p^o)$ for simplicity.

Applying the Langmuir adsorption model, the coverage-dependent chemical potential of surface species can be derived as follows. Given that a surface contains M distinguishable sites, N_i surface species i (i>0) binds on the surface independently, and N_0 free sites remains:

free site	species 1	species 2	species 3	…	species i	…
N_0, q_0	N_1, q_1	N_2, q_2	N_3, q_3	…	N_i, q_i	…

where N_i is the number of surface species or free sites, and q_i is the partition function. If one species occupies one site, we will have

$$M = \sum_{i \geq 0} N_i \quad \text{(S3)}$$

q_0 consists only of high-frequency vibrational modes of substrate atoms, and hence may be treated to be unity. For the localized adsorption of surface species, there is no translational and rotational modes, and q_i (i>0) is constituted only by vibrational modes.[1,2,3]

For such a system, the total partition function can be given by[1]

$$Q(T, M, N_i) = M! \prod_{i \geq 0} \frac{q_i^{N_i}}{N_i!} = \frac{M!}{N_0!} \prod_{i \geq 1} \frac{q_i^{N_i}}{N_i!} \quad \text{(S4)}$$

and on taking logarithm and using Stirling's approximation we can obtain

$$\begin{aligned} \ln Q &= M \ln M - M + \sum_{i \geq 0} N_i \ln q_i - \sum_{i \geq 0} N_i \ln N_i + \sum_{i \geq 0} N_i \\ &= M \ln M + \sum_{i \geq 1} (N_i \ln q_i - N_i \ln N_i) - N_0 \ln N_0 \end{aligned} \quad \text{(S5)}$$

For surface species i, the chemical potential is

$$\mu_i(T, N_i) = -RT \left(\frac{\partial \ln Q}{\partial N_i} \right)_{T, N_j}, \; j \neq 0, j \neq i \quad \text{(S6)}$$

Combining Eqs. (S3), (S5) and (S6), the chemical potential of species i can be obtained:

$$\begin{aligned} \mu_i(T, N_i) &= -RT(\ln q_i - \ln N_i + \ln N_0) \\ &= -RT \ln q_i + RT \ln \frac{N_i}{N_0} \end{aligned} \quad \text{(S7)}$$

Replacing N with surface coverage θ, Eq. (S7) can be further written as:

$$\mu_i(T, \theta_i) = -RT \ln q_i + RT \ln \frac{\theta_i}{\theta_0} \quad \text{(S8)}$$

where θ_i and θ_0 are the surface coverage of species i and free sites. Defining $-RT \ln q_i$ as $\mu_i^o(T)$, i.e. the standard chemical potential of surface species i at temperature T, and replacing free site coverage θ_0 by θ_*, we will obtain the form of Eq. (1) in the main text. The presence of free site coverage reflects the fact that in Langmuir adsorption model surface species need to accommodate at certain adsorption sites, while in gas or liquid phases there are no such counterparts that gaseous molecules or solutes need to be attached to. Note that the chemical potential of free sites is always equal to zero because its partition function is unity, and it will not appear in the equations below.

Similar to gas phase molecules, the temperature dependence of the chemical potential of surface species can be given by:

$$\mu_i(T,\theta_i) = \mu_i^o(0K) + [H_{m,i}(T) - H_{m,i}(0K)] - TS_{m,i}(T) + RT\ln\frac{\theta_i}{\theta_*}$$
$$= \mu_i^o(0K) + \Delta\mu_i(T) + RT\ln\frac{\theta_i}{\theta_*} \tag{S9}$$

where $\mu_i^o(0K)$ is equal to the total energy E_i^{tot} (ZPE corrected), and $\Delta\mu_i(T)$ is the difference between $\mu_i^o(T)$ and $\mu_i^o(0K)$. The thermal correction term $\Delta\mu_i(T)$ is quite different from that of gaseous molecules containing a large entropy term. In contrast, the entropies of simple adsorbed species (e.g. atoms or small molecule fragments) are usually very small, and often neglected in microkinetic treatment. In this work, we use μ_i^o to refer to $\mu_i^o(T)$, and E_i^{tot} to $\mu_i^o(0K)$.

2 Elementary surface processes

In this section, we will employ chemical potentials as key kinetic parameters to express reaction rates and reversibility of several typical elementary surface processes; molecular adsorption, dissociative adsorption, association reaction and their reverse processes.

2.1 Molecular adsorption

For the adsorption of gas phase molecule A,

$$A(g) + * \rightleftharpoons A*$$

the forward reaction rate r_+ can be written according to transition state theory (TST):[4]

$$r_+ = \frac{k_B T}{h}\frac{q^{\neq}}{q_g}\frac{p_A}{p^o}\theta_* \tag{S10}$$

where $q^{\neq}$ is the partition function of the transition state (TS) excluding the vibration between the molecule and the surface (superscript $\neq$ refers to TS), q_g is the partition function of the molecule in the gas phase, and θ_* is the surface coverage of free sites. Combining with Eq. (S1), Eq. (S10) can be rearranged as:

$$r_+ = \frac{k_B T}{h} e^{-\frac{-RT\ln q^{\neq} - (-RT\ln q_g + RT\ln\frac{p_A}{p^o})}{RT}}\theta_* = \frac{k_B T}{h} e^{-\frac{\mu^{\neq,o} - \mu_g}{RT}}\theta_* \tag{S11}$$

where $\mu^{\neq,o}$ is the standard chemical potential of the TS, equal to $-RT\ln q^{\neq}$, and μ_g is the chemical potential of the molecule in the gas phase.

Similarly, the reverse reaction rate r_- is obtained:

$$r_- = \frac{k_B T}{h}\frac{q^{\neq}}{q_{ad}}\theta_A = \frac{k_B T}{h} e^{-\frac{-RT\ln q^{\neq} - (-RT\ln q_{ad} + RT\ln\frac{\theta_A}{\theta_*})}{RT}}\theta_* = \frac{k_B T}{h} e^{-\frac{\mu^{\neq,o} - \mu_{ad}}{RT}}\theta_* \tag{S12}$$

where Eq. (S8) has been used, and q_{ad} and μ_{ad} are the partition function and chemical potential of the molecule on the surface, respectively.

According to the De Donder relation,[5] the net reaction rate is:

$$r = r_+ - r_- = r_+(1 - \frac{r_-}{r_+}) = r_+(1 - z) \tag{S13}$$

where z is the reversibility of the process.[6] From Eqs. (S11) and (S12), the reversibility is equal to:

$$z = e^{\frac{\mu_{ad} - \mu_g}{RT}} \tag{S14}$$

2.2 Dissociative adsorption

With respect to the dissociative adsorption of gas molecule AB,

AB(g) + 2* $\rightleftharpoons$ A* + B*

the forward reaction rate can be given by:

$$r_+ = \frac{k_B T}{h}\frac{q^{\neq}}{q_g}\frac{p_{AB}}{p^o}\theta_*^2 = \frac{k_B T}{h} e^{-\frac{-RT\ln q^{\neq} - (-RT\ln q_g + RT\ln\frac{p_{AB}}{p^o})}{RT}}\theta_*^2 = \frac{k_B T}{h} e^{-\frac{\mu^{\neq,o} - \mu_g}{RT}}\theta_*^2 \tag{S15}$$

Also, the reverse reaction rate is:

$$\begin{aligned} r_- &= \frac{k_B T}{h}\frac{q^{\neq}}{q_A q_B}\theta_A\theta_B = \frac{k_B T}{h} e^{-\frac{-RT\ln q^{\neq} - (-RT\ln q_A + RT\ln\frac{\theta_A}{\theta_*}) - (-RT\ln q_B + RT\ln\frac{\theta_B}{\theta_*})}{RT}}\theta_*^2 \\ &= \frac{k_B T}{h} e^{-\frac{\mu^{\neq,o} - \mu_A - \mu_B}{RT}}\theta_*^2 \end{aligned} \tag{S16}$$

where q_A and q_B are the partition functions of A and B on the surface, respectively, and μ_A and μ_B are the chemical potentials of A and B on the surface, respectively. Thus, the reversibility is

$$z = \frac{r_-}{r_+} = e^{\frac{\mu_A + \mu_B - \mu_g}{RT}} \tag{S17}$$

2.3 Association reaction

For an surface association reaction between adsorbates A and B

A* + B* $\rightleftharpoons$ AB* + *

the forward reaction rate can be written as

$$r_+ = \frac{k_B T}{h}\frac{q^{\neq}}{q_A q_B}\theta_A\theta_B = \frac{k_B T}{h} e^{-\frac{-RT\ln q^{\neq} - (-RT\ln q_A + RT\ln\frac{\theta_A}{\theta_*}) - (-RT\ln q_B + RT\ln\frac{\theta_B}{\theta_*})}{RT}}\theta_*^2$$
$$= \frac{k_B T}{h} e^{-\frac{\mu^{\neq,o} - \mu_A - \mu_B}{RT}}\theta_*^2 \quad \text{(S18)}$$

and the reverse reaction rate is

$$r_- = \frac{k_B T}{h}\frac{q^{\neq}}{q_{AB}}\theta_{AB}\theta_* = \frac{k_B T}{h} e^{-\frac{-RT\ln q^{\neq} - (-RT\ln q_{AB} + RT\ln\frac{\theta_{AB}}{\theta_*})}{RT}}\theta_*^2$$
$$= \frac{k_B T}{h} e^{-\frac{\mu^{\neq,o} - \mu_{AB}}{RT}}\theta_*^2 \quad \text{(S19)}$$

where q_{AB} and μ_{AB} are the partition function and chemical potential of AB on the surface, respectively. Hence, the reversibility is equal to

$$z = \frac{r_-}{r_+} = e^{\frac{\mu_A + \mu_B - \mu_{AB}}{RT}} \quad \text{(S20)}$$

According to the above derivation, we can see the reaction rate of a chemical process on the surfaces can be simply written into a production of three terms: (i) $\frac{k_B T}{h}$, close to 10^{13} at 500 K; (ii) an exponential term of the difference between the chemical potentials of the TS and the reactants; and (iii) the surface coverage term of free sites θ_*^n, where n is the number of surface sites bounded with the reactants (Note that an adsorbed species A* counts one site). It is worth noting that the second term refers to the standard chemical potential $\mu^{\neq,o}$ of the TS with no correction of surface coverages, and the chemical potential μ of the reactant which includes the effect of surface coverage (see Eq. (S9)). As can be seen, the dependence of reaction rates on reactant surface coverages is folded into the coverage-dependent chemical potentials.

3 Some proofs for the main text

3.1 Quasi-equilibrium

In the simple kinetic model proposed in the main text, if $\mu_R^{\neq,o} < \mu_P^{\neq,o}$, we can have the following inequalities according to Eq. (6) in the main text:

$$e^{\frac{\mu_P^{\neq,o}-\mu_R^{\neq,o}}{RT}} = \frac{1-e^{\frac{\mu_P-\mu_I}{RT}}}{e^{\frac{\mu_R-\mu_I}{RT}}-1} > 1 \tag{S21}$$

and further,

$$e^{\frac{\mu_R-\mu_I}{RT}} < 2-e^{\frac{\mu_P-\mu_I}{RT}} < 2 \tag{S22}$$

where the inequality $\mu_R > \mu_I > \mu_P$ (see Eq. (7)) has been used. This inequality leads to $\mu_R \approx \mu_I$ if realizing that a small exponent, say $\mu_R - \mu_I$ being 0.1 eV, will give rise to about 10 of the exponential function at 500 K, indicating that adsorption reaches quasi-equilibrium at steady state.

3.2 Free site coverage on good catalysts

Rearranging Eq. (1) in the main text and applying $\theta_I + \theta_* = 1$ in our simple kinetic model, we can obtain surface free site coverage:

$$\theta_* = \frac{1}{1+e^{\frac{\mu_I-\mu_I^o}{RT}}} \tag{S23}$$

Substituting Eq. (S23) in Eq. (6) in the main text will give the expression of the overall reaction rate, and the derivative of the rate with respect to μ_I^o, $\frac{\partial r}{\partial \mu_I^o}$, must be zero when the rate is a maximum. Thus, take its derivative and rearrange it to obtain:

$$(a_p-1)e^{\frac{\mu_R-\mu_I^o+\mu_P^{\neq,o}}{RT}} + (a_R-1)e^{\frac{\mu_P-\mu_I^o+\mu_R^{\neq,o}}{RT}} + a_P e^{\frac{\mu_P^{\neq,o}}{RT}} + a_R e^{\frac{\mu_R^{\neq,o}}{RT}} = 0 \tag{S24}$$

where a_R and a_P are the slopes of the BEP relations between reaction barriers and enthalpy changes for adsorption and desorption, respectively. Namely, $\mu_R^{\neq,o} \propto a_R \mu_I^o$ and $\mu_P^{\neq,o} \propto a_P \mu_I^o$ and taking derivatives gives $\frac{\partial \mu_R^{\neq,o}}{\partial \mu_I^o} = a_R$ and $\frac{\partial \mu_P^{\neq,o}}{\partial \mu_I^o} = a_P$ which have been used to derive Eq. (S24).

On the other hand, from Eq. (6) we can have:

$$e^{\frac{\mu_I}{RT}} = \frac{e^{\frac{\mu_R+\mu_R^{\neq,o}}{RT}} + e^{\frac{\mu_P+\mu_P^{\neq,o}}{RT}}}{e^{\frac{\mu_R^{\neq,o}}{RT}} + e^{\frac{\mu_P^{\neq,o}}{RT}}} \tag{S25}$$

Thus,

$$e^{\frac{\mu_I-\mu_I^o}{RT}} = \frac{e^{\frac{\mu_R-\mu_I^o+\mu_R^{\neq,o}}{RT}} + e^{\frac{\mu_P-\mu_I^o+\mu_P^{\neq,o}}{RT}}}{e^{\frac{\mu_R^{\neq,o}}{RT}} + e^{\frac{\mu_P^{\neq,o}}{RT}}} \tag{S26}$$

In principle, one can solve Eq. (S24) to obtain the value of μ_I^o, and then substitute it to Eq. (S26), which can be further substituted in Eq. (S23) to acquire θ_*. Here, we will make some assumption to estimate θ_*. It is known that the slopes of BEP relations are normally between 0 and 1, varying with reaction types. As yet, the largest slope is found to be ~0.9 for the dissociation of N_2, NO and CO, TSs being very final state like; the smallest slope is found to be ~0.3 for CH_4→C+4H, NH_3→N+3H and H_2O→O+2H, TSs being very initial state like (see Ref. 7 for detail). Based on this observation, we assume $a_R \approx a_P$, which may not be too bad considering the rest terms in Eq. (S24) are exponentials. Thus, from Eqs. (S24) and (S26) we can obtain:

$$e^{\frac{\mu_I-\mu_I^o}{RT}} \approx \frac{a_R}{1-a_R} \tag{S27}$$

Substituting it in Eq. (S23) will lead to:

$$\theta_* = 1 - a_R \tag{S28}$$

Therefore, based on the simple kinetic model it can be seen that free site coverage θ_* is on the magnitude of 10^{-1} for the optimal catalysts.

3.3 Ammonia synthesis

Ammonia synthesis can be simplified into three main steps:

$$N_2(g) + 2\,* \rightleftharpoons 2\,N^*$$

$$H_2(g) + 2\,* \rightleftharpoons 2\,H^*$$

$$N^* + 3\,H^* \rightleftharpoons NH_3(g)$$

It is known that H_2 adsorption is normally barrierless, and often reaches quasi-equilibrium. Thus, we can have:

$$\mu_{H_2} = 2\mu_{H*} \tag{S29}$$

Also, as long as the reaction proceeds forwardly, from Eq. (7) we have:

$$\mu_{N_2} > 2\mu_{N*} \tag{S30}$$

$$\mu_{N*} + 3\mu_{H*} > \mu_{NH_3} \tag{S31}$$

From these equations, we can obtain the bounds for adsorbed N chemical potential as Eq. (10) in the main text.

References

1 Gupta, M. C. Statistical thermodynamics, Chichester: Wiley, **1990**.

2 Clark, A. The theory of adsorption and catalysis, New York; London : Academic P, **1970**.

3 Hecht, C. E. Statistical thermodynamics and kinetic theory, New York: W. H. Freeman, **1990**.

4 Chorkendorff, I.; Niemantsve, J. W. Concepts of modern catalysis and kinetics, Weinheim, Wiley-VCH, **2003**.

5 (a) De Donder, Th. in "L'Affinité." Gauthier-Villers, Paris, **1927**; (b) Boudart, M. in "Kinetics of Chemical Processes." Prentice-Hall, Englewood Cliffs, NJ, **1968**; (c) Boudart, M. *J. Phys. Chem.* **1983**, *87*, 2786; (d) Holstein, W. L.; Boudart, M. *J. Phys. Chem. B* **1997**, *101*, 9991.

6 (a) Dumesic, J. A. *J. Catal.* **1999**, *185*, 496; (b) Cortright, R. D.; Dumesic, J. A. *Adv. Catal.* **2001**, *46*, 161; (c) Campbell, C. T. *J. Catal.* **2001**, *204*, 520; (d) Dumesic, J. A. *J. Catal.* **2001**, *204*, 525.

7 Cheng, J.; Hu, P.; Ellis, P.; French, S.; Kelly, G.; Lok, C. M. *J. Phys. Chem. C* **2008**, *112*, 1308.
